# Supplementary material for: Integrated Management of Bacterial Wilt and Root-Knot Nematode Diseases in Pepper: Discovery of Phenazine-1-Carboxamide from Pseudomonas aeruginosa W-126
Source: Int J Mol Sci. 2025 Apr 3;26(7):3335. doi: 10.3390/ijms26073335 (PMC11989537; doi:10.3390/ijms26073335)

Supplementary Table S1. Inhibition effect of 209 endophytic bacteria against *R. solanacearum*

Strain	Inhibitory diameter	Strain	Inhibitory diameter	Strain	Inhibitory diameter	Strain	Inhibitory diameter
1	≤5mm	26	≤5mm	51	≤5mm	76	≤5mm
2	≤5mm	27	≤5mm	52	≤5mm	77	≤5mm
3	≤5mm	28	≤5mm	53	≤5mm	78	≤5mm
4	≤5mm	29	≤5mm	54	15.2mm	79	≤5mm
5	≤5mm	30	≤5mm	55	≤5mm	80	≤5mm
6	≤5mm	31	≤5mm	56	≤5mm	81	≤5mm
7	≤5mm	32	≤5mm	57	≤5mm	82	≤5mm
8	≤5mm	33	12.3 mm	58	≤5mm	83	≤5mm
9	≤5mm	34	≤5mm	59	≤5mm	84	≤5mm
10	≤5mm	35	≤5mm	60	≤5mm	85	≤5mm
11	≤5mm	36	≤5mm	61	≤5mm	86	≤5mm
12	≤5mm	37	≤5mm	62	≤5mm	87	≤5mm
13	≤5mm	38	≤5mm	63	≤5mm	88	≤5mm
14	≤5mm	39	≤5mm	64	≤5mm	89	≤5mm
15	≤5mm	40	≤5mm	65	≤5mm	90	≤5mm
16	≤5mm	41	15.2 mm	66	≤5mm	91	≤5mm
17	≤5mm	42	≤5mm	67	≤5mm	92	≤5mm
18	≤5mm	43	≤5mm	68	≤5mm	93	≤5mm
19	≤5mm	44	≤5mm	69	≤5mm	94	≤5mm
20	≤5mm	45	≤5mm	70	≤5mm	95	≤5mm
21	≤5mm	46	≤5mm	71	≤5mm	96	≤5mm
22	≤5mm	47	≤5mm	72	≤5mm	97	≤5mm
23	≤5mm	48	≤5mm	73	≤5mm	98	≤5mm
24	≤5mm	49	≤5mm	74	≤5mm	99	≤5mm
25	≤5mm	50	≤5mm	75	≤5mm	100	≤5mm
Lower connection list							
Upper connection list							
101	≤5mm	129	≤5mm	157	≤5mm	185	≤5mm
102	≤5mm	130	≤5mm	158	≤5mm	186	≤5mm
103	≤5mm	131	≤5mm	159	≤5mm	187	≤5mm
104	≤5mm	132	≤5mm	160	≤5mm	188	≤5mm
105	≤5mm	133	≤5mm	161	≤5mm	189	≤5mm
106	≤5mm	134	≤5mm	162	≤5mm	190	≤5mm
107	≤5mm	135	≤5mm	163	≤5mm	191	≤5mm
108	≤5mm	136	≤5mm	164	≤5mm	192	≤5mm
109	≤5mm	137	≤5mm	165	≤5mm	193	≤5mm
110	≤5mm	138	≤5mm	166	≤5mm	194	≤5mm
111	≤5mm	139	≤5mm	167	≤5mm	195	≤5mm
112	≤5mm	140	≤5mm	168	≤5mm	196	≤5mm

113	≤5mm	141	≤5mm	169	≤5mm	197	≤5mm
114	≤5mm	142	≤5mm	170	≤5mm	198	≤5mm
115	≤5mm	143	≤5mm	171	≤5mm	199	≤5mm
116	≤5mm	144	≤5mm	172	≤5mm	200	≤5mm
117	≤5mm	145	≤5mm	173	≤5mm	201	≤5mm
118	≤5mm	146	≤5mm	174	≤5mm	202	≤5mm
119	≤5mm	147	≤5mm	175	≤5mm	203	≤5mm
120	≤5mm	148	≤5mm	176	≤5mm	204	≤5mm
121	≤5mm	149	≤5mm	177	≤5mm	205	≤5mm
122	≤5mm	150	≤5mm	178	≤5mm	206	≤5mm
123	≤5mm	151	≤5mm	179	≤5mm	207	≤5mm
124	≤5mm	152	≤5mm	180	≤5mm	208	≤5mm
125	25.3mm	153	≤5mm	181	≤5mm	209	≤5mm
126	26.7mm	154	≤5mm	182	≤5mm		
127	≤5mm	155	≤5mm	183	≤5mm		
128	≤5mm	156	≤5mm	184	≤5mm		

Supplementary Table S2. Physiological and biochemical test results of strain W-126

Bacterium	Pyocyanin	Liquefied gelatin	Nitrate reduction	42℃ growth	Oxidase
W-126	+	+	+	+	+

Note: "+" means positive reaction, "-" means negative reaction

Supplementary Table S3. Structure information of PCN analogues

Number	Names	Structures	CAS
1	phenazin-1-ylamine	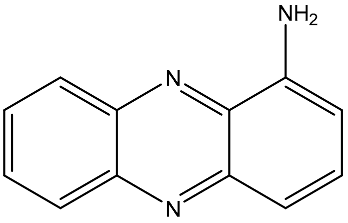	2876-22-4
2	2-Amino-3-phenazinol	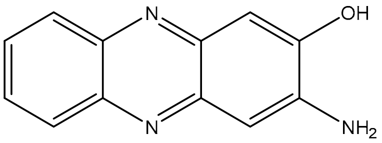	4569-77-1
3	1-Methoxyphenazine	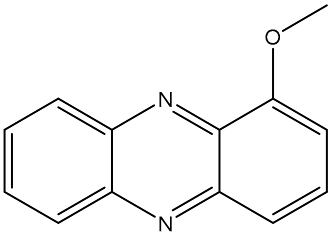	2876-17-7

4	phenazine-1- carboxamide	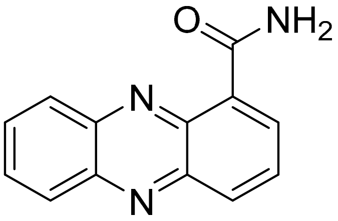	550-89-0
5	Phenazine	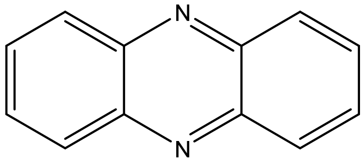	92-82-0
6	phenazin-1-ol	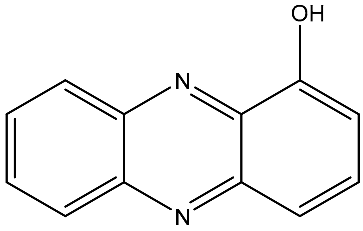	527-71-2
7	Dipyrido3,2-a:2',3'-cphenazine	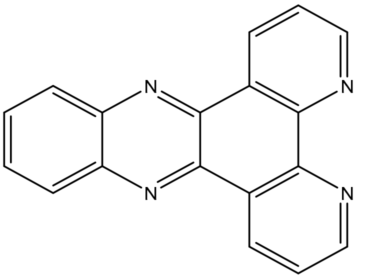	19535-47-8
8	Phenazine-1-carboxylic acid	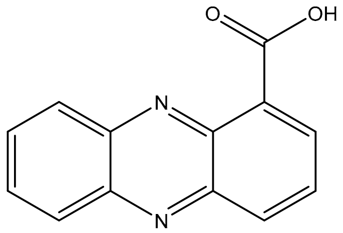	2538-68-3
9	Safranine T	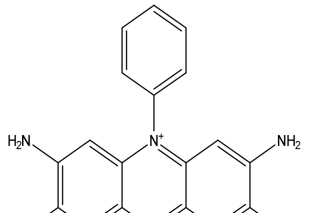	477-73-6
10	Phenazine methosulfate	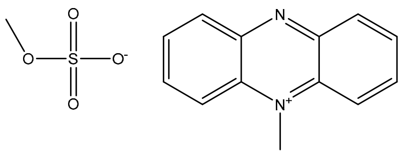	299-11-6

11	Neutral Red	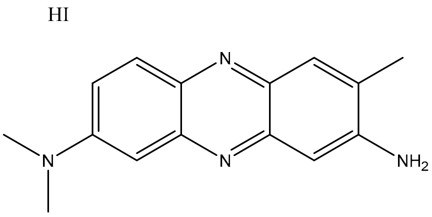	553-24-2
12	2,3-diaminophenazin	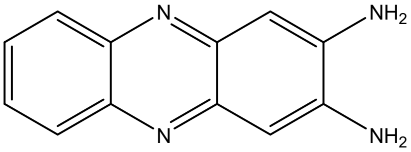	655-86-7
13	Azocarmine G	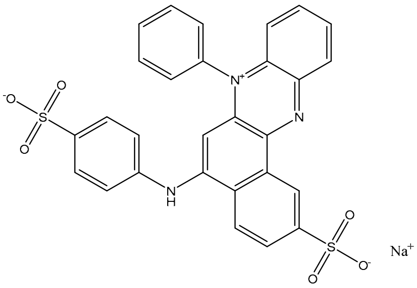	25641-18-3
14	Methylene Violet 3RAX	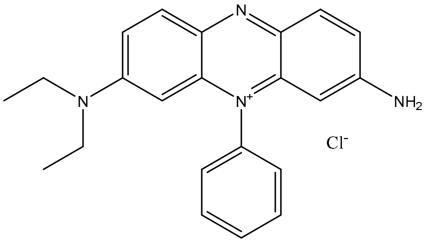	4569-86-2
15	Phenazine ethosulfate	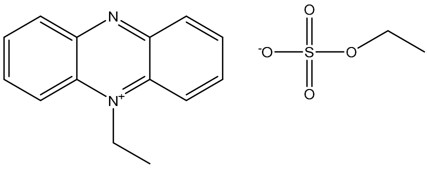	10510-77-7
16	1,6-Phenazinediol	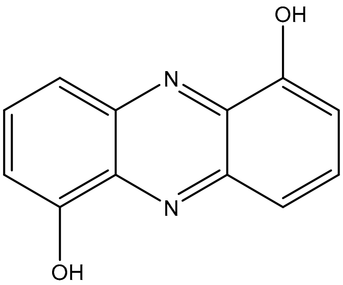	69-48-7
17	Phenazine 5,10-dioxide	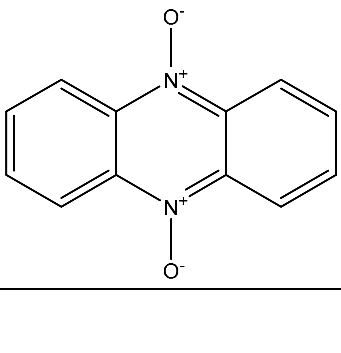	303-83-3

Supplementary Figure S1: Pot experiment result of PCN and other treatments against *R. solanacearum* (**A**) and *M. incognita* (**B**).

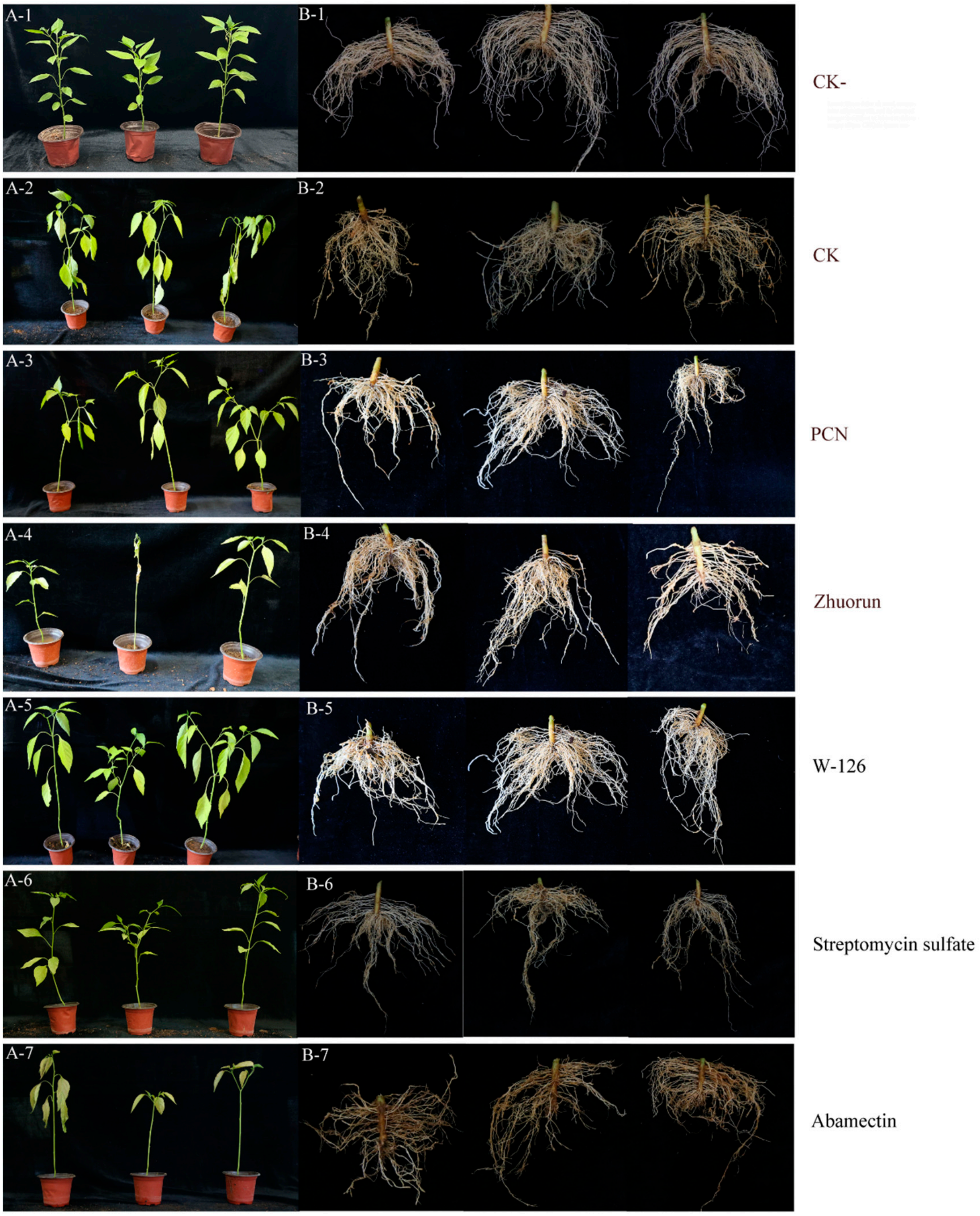

Supplement: Supplementary file 1 [file ijms-26-03335-s001.zip › ijms-3492913-supplementary.pdf]
